# Maternal HIV Infection and Antiretroviral Therapy in Pregnancy: Implications for Vertical Transmission, Fetal Safety, and Long-Term Infant Outcomes

**DOI:** 10.3390/pathogens14080818

**Published:** 2025-08-19

**Authors:** Tudor Fleșeriu, Lorena Elena Meliț, Cristina Oana Mărginean, Adrian Vlad Pop, Anca-Meda Văsieșiu

**Affiliations:** 1 Department of Infectious Diseases, George Emil Palade University of Medicine, Pharmacy, Science and Technology of Targu Mures, 540136 Targu Mures, Romania; tudor.fleseriu@umfst.ro (T.F.); anca-meda.vasiesiu@umfst.ro (A.-M.V.); 2 Doctoral School of Medicine and Pharmacy, George Emil Palade University of Medicine, Pharmacy, Science and Technology of Targu Mures, 540136 Targu Mures, Romania; 3 Infectious Diseases Clinic 1, Mures Clinical County Hospital Mures, 540233 Targu Mures, Romania; infectioase1@spitaljudeteanmures.ro; 4 Department of Pediatrics 2, George Emil Palade University of Medicine, Pharmacy, Science and Technology of Targu Mures, 540136 Targu Mures, Romania; 5 Department of Pediatrics 1, George Emil Palade University of Medicine, Pharmacy, Science and Technology of Targu Mures, 540136 Targu Mures, Romania

**Keywords:** HIV-exposed uninfected children (HEU), mother-to-child transmission (MTCT), antiretroviral therapy (ART), integrase strand transfer inhibitors (INSTIs), pregnancy outcomes, fetal safety, mitochondrial toxicity

## Abstract

HIV mother-to-child transmission (MTCT) continues to pose a significant public health challenge, especially in regions with limited resources, although the worldwide distribution of antiretroviral therapy (ART) has drastically lowered the risk of vertical transmission to even below 1% in some regions. There are still uncertainties regarding the safety of some ART regimens during pregnancy and their longer-term effects on infants who are perinatally exposed to HIV but remain uninfected. This review explores current evidence regarding the interplay between maternal HIV infection, ART during pregnancy, and both maternal and pediatric outcomes. Particular attention is given to the risk/benefit ratio surrounding different drug classes, with integrase inhibitors seeming promising choices in MTCT due to their rapid viral suppression and favorable safety profiles. Meanwhile, regimens containing protease inhibitors or nucleoside reverse transcriptase inhibitors have been linked to some adverse outcomes such as low birth weight, growth restriction, and potential mitochondrial or metabolic disturbances. Although ART remains central in preventing MTCT, a deeper understanding of its effects on fetal development and postnatal health is needed, and it should be thoroughly monitored through future research and longitudinal surveillance.

## 1. Introduction

Human immunodeficiency virus (HIV) was proven to cause acquired immunodeficiency syndrome (AIDS) in 1983, which in fact was reported two years earlier (1981) in the United States of America, mainly in men who have sex with men, thus rapidly becoming a global public health issue [1,2]. HIV infection is a growing clinical concern worldwide, regardless of the patient’s age. Unfortunately, mother-to-child transmission of this infection is one of the major burdens of healthcare systems globally, but especially in sub-Saharan Africa, where more than 90% of newly infected cases occur [3].

It is estimated that around 1.3 million women and girls living with HIV become pregnant each year, and without intervention, the risk of mother-to-child transmission (MTCT) during pregnancy, labor, delivery, or breastfeeding can range from 15% to 45% [4]. However, the implementation of antiretroviral therapy (ART) has been a breakthrough in reducing this risk. Global estimations indicate that 84% (72–98%) of pregnant women living with HIV were receiving ART in 2023, leading to a dramatic decrease in new infections among children [5]. In many regions, the transmission rate has dropped to less than 1% where comprehensive care and ART are widely accessible [6]. Despite these advances, uninterrupted treatment accessibility and access to targeted care remain critical for reaching the global targets in eliminating MTCT, requiring an ongoing integration of HIV interventions into maternal health services, improved access to testing, and continuous ART coverage throughout pregnancy and breastfeeding [6,7]. For example, Botswana’s nationwide prevention of MTCT program, which achieved vertical transmission rates below 5% through universal antenatal HIV testing, same-day ART initiation, and community-based follow-up, demonstrates the impact of such integration [8].

Although the progress in reducing HIV infections in pediatric populations due to prevention programs is definitely incontestable, the aim to eliminate this infection in children by 2015 unfortunately remains only a regrettable clinical failure [9]. Still, in the complete absence of antiretroviral therapy, the mother-to-child transmission rate would reach 40% [10]. Currently, these rates may reach 1% in developing countries, where breastfeeding is unavoidable [11], and even below 1% without breastfeeding [10]. Thus, previous reports highlighted that in South Africa, approximately 50,000 children with HIV are born annually, compared to approximately 190 per year in the USA and 25 per year in the United Kingdom [12]. Older epidemiological studies also confirmed these statistics, proving that the risk of HIV mother-to-child transmission in the absence of any intervention varies between 15% and 30% in Europe and the USA [13,14,15], while within sub-Saharan Africa it might reach even 40% [15,16,17,18]. Most of these transmissions occur within the peripartum period, either in utero or intrapartum [12,19]. The disparities regarding the mother-to-child transmission risks are due to different factors related to the population characteristics, obstetric preferences, and infant feeding patterns [12].

Based on the aforementioned data, it should be recognized that HIV infection in children is a growing pandemic with complex long-term implications. Existing prophylactic protocols recommend antiretroviral treatment for all pregnant and breastfeeding women [20], both to prevent vertical transmission of HIV to the infant and to safeguard the mother’s health [21]. As a result of these strategies, the number of HIV-exposed, uninfected children increases constantly, and according to recent evidence, their overall developmental and postnatal outcomes may be different when compared to their non-exposed counterparts, highlighting a strong need for tailored surveillance [9]. Several studies on HIV-exposed, uninfected cohorts found similar results in terms of increased morbidity and mortality patterns in this subgroup of children [22,23,24]. Thus, a very large study involving 14,110 infants followed-up until the age of 2 years performed in Zimbabwe reported three times higher mortality in the group of children that were perinatally exposed to HIV, compared to their unexposed peers, especially in the first year of life [22]. Another study involving Zambian infants that were followed-up between the age of 9 months and 3 years of age also underlined that HIV-exposed, uninfected infants have a three-times higher mortality rate when compared to the unexposed ones [23]. Similar findings were reported in Uganda, where the mortality rate of the exposed, uninfected group was significantly higher at the age of eighteen months, but not at one or two years of age [24]. Studies that followed these children for a longer period reported contradictory results, indicating no increase in mortality rates in exposed, uninfected children from Rwanda up to the age of 5 years [25], while in those from Gambia that were uninfected, but perinatally exposed to both HIV-1 and -2, the mortality risk was significantly higher below 6 years of age [26]. In terms of morbidity, a more recent study performed on South African HIV-exposed, uninfected children after the implementation of prevention strategies for maternal-to-child transmission pointed out that this group has an increased risk of being admitted for severe infections during the first year of life and of experiencing growth deficits that are refractory to nutritional supplements [27]. The risk for severe respiratory infections was found to be increased in other African studies involving similar groups [28], but gastroenteritis and sepsis were also linked to increased rates of morbidity and mortality in HIV-exposed, uninfected children from India [29] and Europe [30]. Recent studies have consistently demonstrated that HIV-exposed, uninfected children represent a special population with distinct, not yet fully understood vulnerabilities that are associated with higher morbidity and mortality rates compared to their uninfected counterparts. Identifying the precise impact of HIV and maternal/postnatal antiretroviral exposure, as well as the co-factors that negatively influence the well-being of this population, might represent a key component of clinical management in decreasing their risks of severe infections, associated morbidity, and mortality.

This review aims to provide a complex atomistic-to-holistic overview of the HIV pathway and its impact on pregnancy and offspring.

## 2. Search Strategy

Complying with the rules of a narrative review, we searched articles on PubMed^®^ based on the following keywords: HIV, AIDS, mother-to-child HIV transmission, transplacental HIV transmission, ART, HIV-Exposed Uninfected (children), mitochondrial toxicity, Integrase Strand Transfer Inhibitors, Nucleoside Reverse Transcriptase Inhibitors, protease inhibitors, chronic immune activation, and HIV pregnancy. In order to find articles describing niche subjects, we used modern AI searching tools like Elicit.ai. We included studies that focused on assessing mother-to-child HIV transmission, ART, its impact during pregnancy, and its fetal impact. This review required no ethical approval.

## 3. The Bridge Between HIV and Pregnancy

Preconception counselling should represent the first step for all HIV-positive women of reproductive age willing to procreate in order to foresee the most effective methods for preventing vertical transmission of the infection [31]. This group of women should receive appropriate advice regarding reducing the viral load as much as possible before conception to lower the risk of transmission, the treatment of all comorbidities that might increase transmission such as depression, psychiatric or psychosocial conditions, and domestic violence, the choice of the least harmful treatment during pregnancy, and the importance of ART compliance during this period [32,33]. Screening for HIV in pregnancy should also be taken into account, especially in low-income countries with a well-documented increased prevalence of HIV [31]. Moreover, the British HIV Association considers that all women with a positive HIV test should benefit from a psychosocial assessment and continuous support [34].

The frequently reported adverse pregnancy events linked to HIV include stillbirths, miscarriage, intrauterine growth restriction, perinatal mortality, chorioamnionitis, and low birth weight [35]. In terms of maternal infections during pregnancy, HIV was proven to negatively impact the contracting frequency and evolution of human papillomavirus, vulvovaginal candidiasis, herpes simplex, syphilis, bacterial vaginosis, cytomegalovirus, trichomonas vaginalis, hepatitis B and C, toxoplasmosis, bacterial pneumonia and urinary tract infections, and specific HIV-associated opportunistic infections like *Pneumocystis jirovecii* pneumonia and tuberculosis [36]. Indeed, the increased use of antiretroviral therapies noticeably suppresses the virological and immunological risks in women living with HIV, but their long-term prognosis does not necessarily reflect that of the uninfected ones [37,38,39], most likely due to the overall growth and cardiovascular and metabolic disturbances [40]. In fact, a recent study that compared 614 HIV-positive to 390 HIV-negative pregnant women concluded that the risk of adverse outcomes during pregnancy remains two to three times higher in the HIV-positive group on antiretroviral therapy when compared to the HIV-negative group [41]. Thus, more recent studies have continued to ascertain the increased risk among HIV-positive women of preterm delivery or having small-for-gestational-age infants with a low birth weight or significantly lower than unexposed controls [37,38,42,43]. A recent study performed on a large sample of HIV-positive pregnant women and their exposed newborns found that 22.5% of the newborns had a low birth weight, 22% were born preterm, 18% were small for gestational age, and 4% had a very low birth weight [44]. Still, the results from different multicentric studies remain controversial, most likely due to other factors that might influence pregnancy outcomes such as maternal age and body mass index, socio-economic status, ethnicity, substance abuse, smoking, multiple gestation, etc. [45,46,47].

One very important incriminated pathogenic pathway resides in the chronic immune activation associated with HIV infection [48]. It has already been proven that seropositive pregnant women express higher concentrations of pro- and anti-inflammatory cytokines in both their plasma and the cord blood [49,50]. A maternal pro-inflammatory state has been linked to various negative outcomes in their offspring, especially neurological disorders, and influences overall development, as reflected in different height and weight gain patterns [51,52].

Studies investigating neurologic disorders in children born to HIV-infected mothers have established a connection between the higher levels of inflammatory mediators and their involvement in microglial dysfunction. As the brain’s glial cells, microglia have a central role in brain maturation, synaptic modeling, and overall neurodevelopment [53,54,55]. Studies show that the upregulation of microglial activation genes, especially the M2-microglial activation module, as seen in inflammatory responses, lowers the expression of neural activity genes, thus linking innate immunologic stimulation to neurodevelopmental alterations [56,57]. In the case of children born to HIV-infected mothers, different cytokine patterns have been observed, with some studies describing persistent overstimulation of interleukin-4 (IL-4) up to 12 years of age, a key stimulator of the M2-microglial activation module [58,59,60].

During pregnancy, in order to prevent fetal rejection reactions, maternal immunity physiologically inhibits cellular-mediated responses and boosts humoral responses, thus shifting from T-helper 1 (Th1) stimuli to Th2 stimuli predominance [61]. However, in women living with HIV, this phenomenon can differ, resulting in increased levels of both Th1 and Th2-mediated cytokines, since both these cell types possess the C-X-C motif chemokine receptor 4 (CXCR4) and the chemokine (C-C motif) receptor 5 (CCR5) [62]. The increase in Th1 stimuli during pregnancy has been linked to negative perinatal outcomes. There are many proposed pathophysiological pathways, including the fact that preterm births have been correlated to higher tumor necrosis factor α (TNF-α) levels [63], a pro-inflammatory environment may disrupt normal prostaglandin activity, thus heightening the risk of premature membrane rupture [64], and interleukin-2 (IL-2) as well as interleukin-12p70 (IL-12p70) were found in higher concentrations in small-for-gestational-age newborns [65], among other factors. The reasoning behind overall fetal growth restriction in this maternal context seems to lie at the intersection of many pathological processes involving the placenta which are influenced by inflammatory triggers, including aberrant spiral artery remodeling, which combined with oxidative stress, endothelial dysfunction, and microthrombosis may lead to insufficient oxygen and nutrient flow [66,67].

As in their mothers, a pro-inflammatory state has been observed in these children, with studies describing increased levels of interleukin-8 (IL-8) and interleukin-6 (IL-6) inside the cord blood when compared to unexposed peers [50]. It is worth mentioning that IL-8 does not cross the placenta, thus suggesting its fetal origin [68]. In the case of IL-6, however, whether it permeates the placenta under special conditions like inflammation, or not, is not yet clear, although more recent studies demonstrate the latter [69,70]. Regardless of its origin, it is well established that high fetal IL-6 levels, influencing overall growth, neuro-endocrine functions, and bone maturation, have been linked to various cardiovascular risks [69,70,71,72]. These outcomes can be justified by the fact that increased fetal IL-6 levels suppress insulin-like growth hormone (IGF-1), which is essential for proper intrauterine growth and maturation [72,73].

Recently, it was highlighted that higher levels of soluble endoglin, an antiangiogenic factor which acts by inhibiting the transforming growth factor, might be involved in preterm delivery, decreased levels of placental growth factor and subsequent risks of small-for-gestational-age infants, and stillbirths in HIV-positive women on antiretroviral therapy [74]. Similar findings were revealed in a study on 24 HIV-positive pregnant women, which pointed out reduced mitochondrial DNA (mtDNA) content, increased placental apoptosis, and oxidative stress, but failed to identify a correlation with the risk of small for gestational age or preterm delivery [75].

Researchers began to question the adverse effects of antiretroviral therapy on pregnancy outcomes. Thus, studies that focused on this topic underlined that in addition to the infection’s adverse effects on pregnancy, antiretroviral therapy during conception is associated with an increased risk of adverse pregnancy effects, including the risk of small-for-gestational-age infants [43,76,77] and even spontaneous abortion or stillbirths [78], although its benefits overwhelmingly outweigh these risks regarding both maternal and fetal outcomes [79]. Regarding the stratification of this risk depending on antiretroviral class, it was suggested that protease inhibitors are involved in the increased risk of preterm delivery [38,76,77,80]. Although the highest risk of preterm delivery was found in women who conceived while under lopinavir/ritonavir regimens, regardless of the baseline CD4 count, other protease inhibitors were also suggested to increase the risk of preterm delivery in the setting of CD4 counts under 350 cells/μL [77]. Likewise, tenofovir disoproxil fumarate is associated with a similar risk of adverse pregnancy outcomes [81].

## 4. The Trialogue Between HIV, Antiretroviral Therapy, and the Child

The benefits of antiretroviral therapy in preventing HIV mother-to-child transmission are unquestionable, but each drug is associated with a possible negative effect on the fetus/newborn [44]. The most common adverse effects reported in the literature include hepatic, hematological, and mitochondrial alterations, newborn mortality, preterm birth, congenital malformations, intrauterine growth restriction, low birth weight, and viral resistance [82,83,84,85,86]. Nevertheless, recent evidence suggests that the benefits of antiretroviral therapy easily surpass its risks, highlighting the importance of this therapy for HIV control in mother-to-child transmission [44].

### 4.1. Metabolic Alterations

Mitochondrial impairment was suggested decades ago to be the most important mechanism involved in the poorer outcome of HIV-exposed, uninfected infants born to mothers who received antiretroviral therapy. Therefore, older studies reported antiretroviral therapy to cause mtDNA mutations, changes in mtDNA levels, or alterations in the histological morphology of mitochondria [87,88]. In fact, nucleoside reverse transcriptase inhibitors, especially zidovudine, which is the most frequently used drug during pregnancy, representing the cornerstone of regimens for both HIV-positive pregnant women and their exposed offspring, seem to be the most harmful due to their ability to bind to mitochondrial gamma-DNA polymerase [89,90], a crucial enzyme responsible for the replication and repair of the mitochondrial DNA, and alter its replication [91,92]. Thus, nucleoside reverse transcriptase inhibitors will eventually decrease the copy number and alter the quality of the mitochondrial DNA genome, augmenting reactive oxygen species production [91,92]. In addition, all of these effects of the aforementioned drugs on mitochondria are augmented by HIV itself. In terms of uninfected children exposed to nucleoside reverse transcriptase inhibitors in utero, it was proven that they have a 0.26% higher incidence of presenting mitochondrial dysfunction in spite of minor clinical findings [93]. The question remains whether these documented mitochondrial alterations have a negative long-term impact or not.

Lipid metabolism parameters are not spared by the negative impact of HIV and antiretroviral therapy. Hypertriglyceridemia is not uncommon in patients with HIV infection and protease inhibitor treatment, and it is associated with microbial translocation and high levels of type I interferons [94,95]. It was suggested that protease inhibitors impact fetal lipid metabolism based on decreased levels of cytochrome P450 enzyme 4A (CYP4A) and cytochrome P450 enzyme 2B6 (CYP2B6) eicosanoids encountered in infants exposed to HIV and antiretroviral therapy, since the protease inhibitors are known to reduce the activity of the cytochrome P450 enzyme system (CYP450) [96,97]. It was also suggested that certain metabolites might have a role in modulating immune responses before birth based on a positive correlation between lipid peroxidation products and C-X-C Motif Chemokine Ligand 10 (CXCL10) [97], a well-known stimulating gene of interferon, formerly associated with mitochondrial stress in animal models [98]. Furthermore, the increased level of triglycerides described above enables the activation of endoplasmic reticulum stress’s unfolded protein response, resulting in a downstream of pro-inflammatory proteins [97]. Moreover, infants previously exposed to HIV infection and antiretroviral therapy, although not infected, present reduced levels of arachidonic acid-derived CYP450 metabolites, which are well known for their potent anti-inflammatory effects; thus, the compensatory homeostatic mechanisms could definitely be impaired in these infants [97].

**Figure 1:**
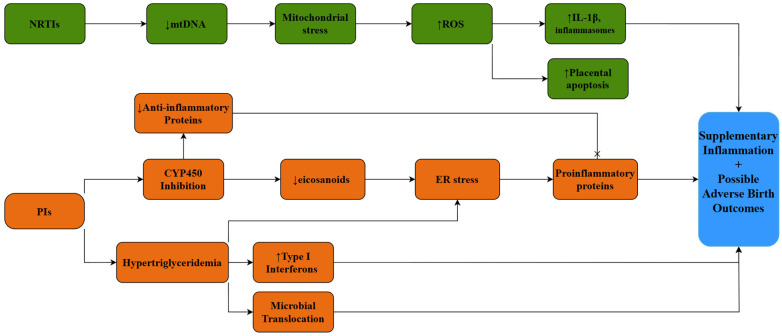
Possible side effects of antiretroviral therapy during pregnancy. Legend: CYP450: Cytochrome P450 Enzyme System; ER: endoplasmic reticulum; IL-1β: interleukin-1 Beta; mtDNA: mitochondrial DNA; NRTIs: nucleoside reverse transcriptase inhibitors; PIs: protease inhibitors; ROS: reactive oxygen species. →: indicates a relationship of cause and effect; ×: indicates the cessation of a stimulus.

A study that assessed the mitochondrial and apoptotic parameters of HIV-positive mothers and their infants, comparing them to those of uninfected/unexposed controls, revealed that certain mitochondrial parameters were significantly reduced in infected mothers and their newborns, such as maternal and fetal mitochondrial synthesis, the levels of mitochondrial DNA in newborns, and the function glycerol-3-phosphate + complex III in the mothers [99]. The authors also pointed out that the apoptosis process was augmented in infected mothers, but not in their children. All of these findings suggest that mitochondrial impairment due to both antiretroviral therapy and HIV itself might represent an important puzzle piece in the immune–metabolic link with a major negative impact on pregnancy outcomes and exposed uninfected newborns (Figure 1).

### 4.2. Immunological Changes

The relationship between the metabolic and cellular immune response is incontestable during infections. The nutrient-sensing receptors located in the endoplasmic reticulum sense the impairment of nutrient levels and subsequently activate the nuclear factor kappa-light-chain enhancer of activated B cells along with the c-Jun N-terminal kinase pathways [100]. Additionally, mitochondrial stress generates reactive oxygen species, which induce the production of interleukin 1 B and inflammasomes [101]. Even in the absence of the infection, pregnancy itself represents a stressful condition which imposes major nutrient consumption and an increase in maternal and fetal metabolism [102]. These peculiarities were proven to heighten the levels of pro-inflammatory cytokines in the placenta within the setting of intrauterine restricted growth as a consequence of decreased nutrient levels [103,104]. Furthermore, as already mentioned in HIV-positive pregnant women, zidovudine, as part of prevention regimens for mother-to-child transmission, is a supplementary trigger of mitochondrial stress in infants, proven through the decreased mitochondrial DNA and acyl-carnitine levels in infants who were exposed to both HIV infection and antiretroviral therapy [93,99,105,106].

**Figure 2:**
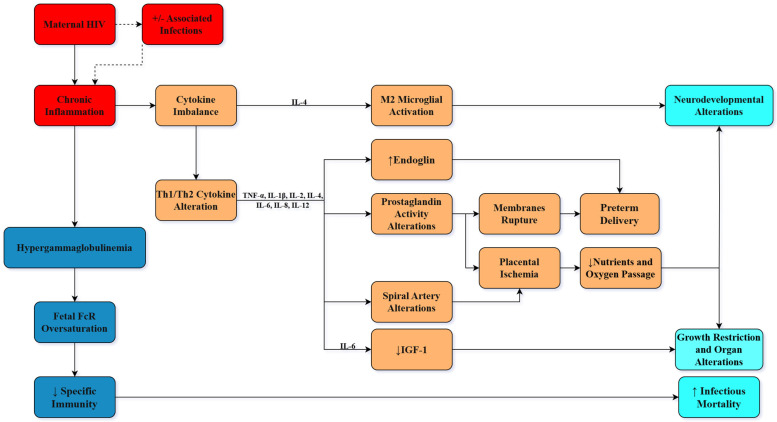
The effects of maternal HIV-associated pro-inflammatory status on pregnancy outcomes. Legend: FcR: Fc receptor; IGF-1: insulin-like growth factor 1; IL: interleukin; Th1/Th2: T-helper cell type 1/2; TNF-α: tumor necrosis factor alpha. →: indicates a relationship of cause and effect; ⤑: indicates an optional relationship of cause and effect.

Metabolic homeostasis represents the key to a competent, highly effective postnatal immune system. Schoeman et al. proved that exposure to HIV infection and long-term antiretroviral therapy during the intrauterine life results in immune malfunction in the postnatal period, which is essential for the development and maintenance of a tolerant immune core during fetal development [97]. The potency of the infant’s immune responses is compromised due to cellular stress triggered by viruses and pharmacological interventions, leading to the overexpression of pro-inflammatory immune responses, subsequent poor pregnancy outcomes, and altered postnatal immune competence [97]. Although it may seem odd, the persistent low-level immune activation and subsequent continuous inflammation are the main determinants of increased susceptibility to infections, as previously suggested in patients with obesity, regardless of their age [107,108]. Recent studies show that these newborns possess less efficient specific immune responses, hinting at an impaired IgG antibody placental transfer [109,110]. Martinez et al. provided evidence that due to the maternal hypergammaglobulinemia, neonatal Fc receptors become oversaturated, reducing the transfer efficiency of specific IgG molecules and thus lowering the passive immunity, which is crucial within the first few months of life [111]. Although these differences are noticeable and the pathogenesis behind them is somewhat understood, it is important to emphasize that much of the reviewed literature studied subtle, subclinical changes, without implying that complications always occur (Figure 2).

### 4.3. ART and the Placenta

The success of ART in lowering MTCT is undeniable, but the efficacy, pharmacokinetics, and fetal and maternal safety of these drugs during pregnancy are still not thoroughly studied, and their use is frequently based on limited evidence rather than robust clinical trial data [112]. Moreover, both placental development and fetal programming might be affected by ART, resulting in different susceptibilities to pathologies later in adulthood [113]. In spite of all of these mysteries, ART remains the cornerstone of care for HIV-positive pregnant women in terms of reducing MTCT transmission, since increased maternal viral load is the most prominent independent risk for MTCT in utero and intrapartum. Other risk factors that increase MTCT include viral phenotypes, advanced HIV-1-associated conditions/AIDS, prolonged amniotic membrane rupture, maternal co-infections, and the presence of different polymorphisms of genes responsible for immune responses or virus entry [114]. Certain studies also highlighted a higher risk of MTCT in mothers who acquire the infection during pregnancy as compared to those diagnosed with HIV before conception [115,116].

The placenta, probably the most important ‘organ’ during pregnancy, might be considered a modulator of MTCT, playing a dual role as both a barrier and sanctuary for HIV. The placenta acts as a barrier by reducing viral passage, but does not entirely prevent MTCT, as the last 14 days of pregnancy carry the highest risk of transmission [117,118]. Additionally, it was proven that HIV infects and replicates within the placental trophoblasts, but with less efficacy compared to CD4+ lymphocytes, since these cells may sometimes express low levels of CCR5 and CXCR4 on their surface, thus making them a viral target [119,120,121]. Other compounds that actively contribute to the reduction in placental viral load include macrophage inflammatory protein-1 beta, placental chemokine (C-C motif) ligand 5, leukemia inhibitory factor, and syncytin [122,123]. Contrariwise, cytokines like interleukin-1,-8 and tumor necrosis factor alpha, synthesized by the placenta or produced during certain infections, were proven to augment transplacental HIV transmission [121,123]. Based on all of these facts, it is clear that the placenta is a perfect host for HIV, while the virus is less susceptible to ART within the trophoblasts when compared to free-living viral particles. Moreover, ART is not able to completely eradicate the infection inside the placenta, which can reactivate after the discontinuation of therapy [122].

### 4.4. ART and Pregnancy

Commonly, ART is meant to decrease viral loads in infected hosts, but during pregnancy, its additional aim is to offer pre-exposure fetal prophylaxis despite the potential harmful effects on the fetus [112,124]. Although the evaluation of antiretroviral placental transport is performed by measuring drug concentrations inside the umbilical cord blood and maternal plasma at the time of delivery, these measurements provide no information regarding the time-dependent pharmacokinetics of these drugs [113]. Thus, in order to provide the highest prophylactic level possible for the unborn baby, the World Health Organization (WHO) recommends the initiation of triple therapy in all pregnant and breastfeeding women, regardless of their clinical stage, viral load, or CD4 cell count, and to maintain this therapy not only during pregnancy, but also during labor, breastfeeding, and later in life [20]. Several factors should be taken into account when choosing the most appropriate drugs for this category of women: whether the woman is treatment-naïve or has been treated priorly with antiretrovirals, the presence of co-morbidities such as hepatitis B or C, and drug toxicity [31]. The most recommended regimens include the use of two nucleosides such as tenofovir/emtricitabine and abacavir/lamivudine or zidovudine/lamivudine, taking into consideration prior antiretrovirals regimens or the resistance profile if available, the interactions with the third antiretroviral agent, the administration frequency, the side effect profiles, and the risk of prematurity or neonatal death that were reported in recent trials for the tenofovir/emtricitabine and lopinavir regimens [79,125].

The priority in HIV-positive pregnant women is to achieve the lowest viral load possible before delivery by initiating antiretroviral treatment as soon as possible, considering the presence of nausea and vomiting during the first trimester of pregnancy as well as the teratogenicity potential of antiretrovirals [31]. Thus, if the pregnant woman’s status allows for it, treatment can be delayed until the end of the first trimester of pregnancy, with the condition of committing to ART treatment by 18–20 weeks of gestation [34]. Based on all of the aforementioned facts, the British HIV Association guidelines advise to start antiretroviral: (1) as early as possible during pregnancy; (2) no later than the 24th week of pregnancy; (3) during the first trimester when the baseline viral load is higher than 100,000 HIV RNA copies/mL and/or when the CD4 cell count is below 200 cells/mm^3^ [34]. If the pregnant woman presents after week 28 of pregnancy and has a viral load beyond 100,000 HIV RNA copies/mL or if the viral load is unknown, the recommendation is to include raltegravir in the three- or four-drug regimen since it is proven to trigger more rapid viral load suppression compared to other antiretrovirals [126]. These recommendations have been issued in order to reduce the embryo’s ART exposure as much as possible, while also preventing transmission in the later stages of pregnancy. However, according to the U.S. Department of Health and Human Services (DHHS) guidelines, pregnant women should start ART as soon as possible, regardless of virological (HIV RNA load) or immunological (CD4 count) parameters [124].

A relatively recent review pointed out a current shift in terms of HIV treatment during pregnancy from protease inhibitors to integrase strand transfer inhibitors [127]. These drugs were proven to be superior to protease inhibitors in terms of their viral load suppression rapidity, and due to the fact that they do not possess the problem of viral escape, the need to introduce an additional antiretroviral agent such as cobicistat or ritonavir is eradicated [127]. The first studies performed on HIV-pregnant women using raltegravir suggested that it successfully decreased their viral loads, resulting in no MTCT of HIV, while also not being associated with any biological adverse effects or fetal abnormalities despite its high fetal cord blood concentrations [128,129]. In spite of the high variability regarding the concentrations of raltegravir in both pregnant and non-pregnant adults, studies pointed out that the viral load decreased below 400 copies/mL in 92% of the pregnant women, with no cases of vertical transmission, and 95% of the non-pregnant adults achieved viral suppression by the fourth week of therapy [130,131]. Moreover, a longitudinal study found a viral suppression of <50 copies/mL in 86% of the patients, with no vertical transmission or fetal malformations at the time of delivery, nor have there been reported any adverse events at 6 years after the initiation of the study [132]. Another important drug belonging to the class of integrase strand transferase inhibitors is dolutegravir, which is the most preferred third agent in certain guidelines, while other guidelines recommend a choice between this agent and raltegravir [127].

The preference for dolutegravir instead of raltegravir is a consequence of the increased resistance barrier. Overall, studies involving dolutegravir indicated similar if not better outcomes when compared to raltegravir in terms of viral suppression, reporting viral loads below 50 copies/mL at the time of delivery in more than 90% of the participating women [133,134,135]. Studies including small populations of pregnant women suggested that dolutegravir might increase the risk of neural tube defects in newborns [136,137]. Thus, for some time, most guidelines advised against using dolutegravir during the first trimester of pregnancy, and especially in the first 6 weeks of pregnancy due to these concerns [127]. Broader ongoing studies have demonstrated a small difference between dolutegravir and non-dolutegravir regimens, but the final conclusion regarding the correlation between neural tube defects and dolutegravir usage is still under investigation [138]. However, in updated guidelines, dolutegravir was proven to be a safe option even in the first weeks of pregnancy [139,140]. Bictegravir, the newest addition to the integrase strand transferase inhibitor class, has also shown similar virological suppression capabilities, as well as a good safety profile in pregnant women, while currently being recommended as a safe choice by the Department of Health and Human Services guidelines, although more evidence must be provided [141,142,143,144].

Currently, the British HIV Association guidelines (2025), as well as the WHO consolidated guidelines (2021), recommend dolutegravir-based regimens as first-line ART in pregnancy [34,124,145], while in their 2025 updated guidelines, the DHHS started recognizing bictegravir-based regimens as the preferred ART for gestating women. Therefore, bictegravir and dolutegravir remain the best choices of antiretroviral agents during pregnancy, being proven to rapidly suppress the viral load in pregnant women with >100,000 HIV RNA copies/mL or in those with unknown virological status, as well as in those that do not respond to other antiretrovirals or who present with uncontrolled HIV infection during the third trimester of pregnancy [124,127].

We compiled the information regarding the efficacy and safety of integrase inhibitors during pregnancy, as shown in Table 1. Moreover, we also highlighted potential adverse fetal outcomes associated with different ART drug classes, as shown in Table 2.

## 5. Clinical Implications for Practice

HIV-exposed uninfected children are a unique clinical group that need continuous health monitoring, extending beyond the neonatal period. Clinicians should implement a structured follow-up lifelong plan. This plan should prioritize growth monitoring, developmental assessments, and timely evaluations for infectious illnesses, particularly during the first few years of life. ART selection in pregnancy should be guided by up-to-date safety data: integrase strand transfer inhibitors such as dolutegravir and raltegravir offer rapid viral suppression and are generally well tolerated, although the regimen choice must consider gestational timing, maternal comorbidities, and potential teratogenic risks (Table 3). Protease inhibitor-based regimens should be used with awareness of their association with preterm birth and low birth weight. For healthcare practitioners, the main priorities are as follows: begin antiretroviral therapy as early as safely possible during pregnancy, ensure that the mother maintains viral suppression throughout her pregnancy and breastfeeding, and provide comprehensive care that brings together obstetric, infectious disease, and pediatric expertise to enhance outcomes for both the mother and the infant.

## 6. Conclusions

Mother-to-child transmission of HIV remains a critical global health issue, with substantial progress achieved through the implementation of ART as well as other practices aiming to minimize the horizontal transmission risk. HIV mother-to-child transmission has significantly declined in settings where comprehensive care is available, yet disparities persist, particularly in low-resource areas, like South Africa. While ART effectively prevents MTCT, there is growing evidence hinting at the fact that HIV-exposed, uninfected children are characterized by increased morbidity and mortality, highlighting the possible need for lifelong health monitoring and tailored intervention strategies.

HIV infection can complicate pregnancy by increasing the likelihood of adverse outcomes such as preterm birth, low birth weight, and stillbirths. While ART plays a fundamental role in reducing transmission, certain antiretroviral drugs have been associated with metabolic, mitochondrial, and immunological disturbances. The selection of an appropriate ART regimen is crucial for successfully controlling viral suppression in order to prevent MTCT while also ensuring fetal safety. Many studies described pregnancy complications or alterations in perinatal outcomes attributed to protease inhibitors and nucleoside reverse transcriptase inhibitors, but integrase inhibitors are emerging as a promising option regarding the safety profile of the aforementioned alterations and offer faster and stronger viral load control, despite a few ongoing concerns regarding teratogenicity. Although integrase strand transfer inhibitors seem to result in better viral suppression, the data regarding their long-term safety are scarce when compared to protease inhibitors and nucleoside reverse transcriptase inhibitors.

Future research should focus on the long-term effects of ART on both mothers and their offspring, optimizing treatment regimens to enhance maternal and fetal well-being. Policymakers and healthcare providers must ensure widespread access to ART, integrated maternal health services, and continuous monitoring of HIV-exposed infants to mitigate long-term health risks. The ultimate goal remains the complete eradication of pediatric HIV while minimizing the adverse effects of both the virus and antiretroviral treatment on maternal and child health.

The main limitation of this review is represented by its narrative (non-systematic) nature, which exhibits variability in terms of the quality and design of the included studies. Moreover, we must acknowledge the regional disparities regarding ART availability, which might also result in relative objectivity regarding the impact of ART on pregnancy.

Indeed, more robust surveillance and longitudinal studies are needed to quantify the impact of ART on fetal development, childhood health, and maternal well-being. Collaboration between governmental, non-governmental organizations, and research institutions is essential in refining treatment protocols and enhancing care accessibility in low-resource settings. Addressing socio-economic and healthcare disparities remains imperative in improving outcomes for both HIV-positive mothers and their offspring. By fostering a multidimensional approach that combines medical, social, and policy-driven efforts, the global community can move closer to the priority of eliminating pediatric HIV while ensuring comprehensive and sustainable healthcare solutions.

## Figures and Tables

**Table 1:** Comparative efficacy and safety of integrase inhibitors during pregnancy.

Drug	Key Benefits	Potential Risks	Summary of Findings
Raltegravir	Rapid viral load suppression; Safe in all trimesters; No fetal malformations reported	High pharmacokinetic variability	Suppresses viral load < 400 copies/mL in 92% of pregnant women
Dolutegravir	Stronger resistance barrier; Faster viral suppression; WHO and BHIVA recommended as 1st-line therapy	Early concerns over neural tube defects in the 1st trimester	>90% achieved VL < 50 copies/mL at delivery; newer evidence confirms safety in early pregnancy
Bictegravir	High virological efficacy; Good safety profile per emerging data; DHHS recommends as 1st-line therapy	Limited pregnancy-specific data; Not yet widely adopted	Similar suppression and safety to dolutegravir

Legend: BHIVA: British HIV Association (Guidelines); DHHS: Department of Health and Human Services (Guidelines); VL: viral load; WHO: World Health Organization (Guidelines).

**Table 2:** Adverse fetal outcomes associated with different ART drug classes.

ART Class	Representative Drugs	Adverse Fetal Outcomes	Notes
Nucleoside Reverse Transcriptase Inhibitors (NRTIs)	Zidovudine, Lamivudine, Abacavir, Tenofovir	Mitochondrial dysfunction (Decreases mitochondrial DNA content, increases reactive oxygen species production) Growth restriction Preterm birth Low birth weight	Zidovudine interferes with mitochondrial γ-DNA polymerase, leading to impaired replication and oxidative stress
Protease Inhibitors (PIs)	Lopinavir/ritonavir, Atazanavir	Preterm birth Small for gestational age Low birth weight Hypertriglyceridemia	Lopinavir/ritonavir linked to increased prematurity risk, especially when antiretroviral therapy is initiated at conception
Integrase Strand Transfer Inhibitors (INSTIs)	Raltegravir, Dolutegravir, Bictegravir	(Early concerns) Neural tube defects with dolutegravir Overall favorable profile High efficacy in viral suppression	Dolutegravir linked to neural tube defects in early reports, but recent data support its safety

**Table 3:** First-line recommended ART regimens in pregnant women.

Guideline	BHIVA (2025)	DHHS (2025)	WHO (2021)
First-line regimens in pregnancy	INSTI: DTG Backbone: TDF + FTC	INSTI: BIC Backbone: TAF + FTC	INSTI: DTG Backbone: TDF + 3TC/FTC

Legend: BHIVA: British HIV Association; DHHS—U.S. Department of Health and Human Services; WHO: World Health Organization; INSTI: integrase strand transfer inhibitor; DTG: dolutegravir; BIC: bictegravir: TDF: tenofovir disoproxil fumarate; TAF: tenofovir alafenamide; FTC: emtricitabine; 3TC: lamivudine.

## Data Availability

No new data were created or analyzed in this study.

## Abbreviations

The following abbreviations are used in this manuscript:

3TC	Lamivudine
AIDS	Acquired immunodeficiency syndrome
ART	Antiretroviral therapy
BHIVA	British HIV Association
BIC	Bictegravir
CCR5	Chemokine (C-C motif) Receptor 5
CD4+	Cluster of Differentiation 4 Positive
CXCL10	C-X-C Motif Chemokine Ligand 10
CXCR4	C-X-C Motif Chemokine Receptor 4
CYP2B6	Cytochrome P450 Enzyme 2B6
CYP450	Cytochrome P450 Enzyme System
CYP4A	Cytochrome P450 Enzyme 4A
DHHS	U.S. Department of Health and Human Services
DTG	Dolutegravir
ER	Endoplasmic reticulum
FTC	Emtricitabine
HEU	HIV-exposed uninfected (children)
HIV	Human immunodeficiency virus
IGF-1	Insulin-like growth hormone
IL-1β	Interleukin-1 Beta
IL-2	Interleukin-2
IL-4	Interleukin-4
IL-6	Interleukin-6
IL-8	Interleukin-8
IL-12p70	Interleukin-12p70
INSTIs	Integrase strand transfer inhibitors
MTCT	Mother-to-child transmission
mtDNA	Mitochondrial DNA
NRTIs	Nucleoside reverse transcriptase inhibitors
PIs	Protease inhibitors
ROS	Reactive oxygen species
TAF	Tenofovir alafenamide
TDF	Tenofovir disoproxil fumarate
TNF-α	Tumor necrosis factor alpha
WHO	World Health Organization
